# Comparative Evaluation of Tensile Bond Strength of Poly Ether Ether Ketone (PEEK) and Zirconia Copings Using Resin Cement with or without Adhesive: An In Vitro Study

**DOI:** 10.3390/ma15124167

**Published:** 2022-06-12

**Authors:** Nimisha Kakkad, Naveen S. Yadav, Puja Hazari, Shweta Narwani, Kirti Somkuwar, Sakeenabi Basha, Varsha Verma, Suraj Arora, Omir Aldowah, Artak Heboyan, Mohmed Isaqali Karobari

**Affiliations:** 1 Department of Prosthodontics Crown & Bridge and Implantology, Peoples Dental Academy, Peoples University, Bhopal 463027, India; naveensyadav@gmail.com (N.S.Y.); drhazaripooja@gmail.com (P.H.); drshwetanarwani@gmail.com (S.N.); drkirtisomkuwar@gmail.com (K.S.); varshaverma1987@gmail.com (V.V.); 2 Department of Community Dentistry, Faculty of Dentistry, Taif University, P.O. Box 11099, Taif 21944, Saudi Arabia; sakeena@tudent.edu.sa; 3 Department of Restorative Dental Sciences, King Khalid University, P.O. Box 960, Abha 61421, Saudi Arabia; sprakash@kku.edu.sa; 4 Prosthetic Dental Science Department, Faculty of Dentistry, Najran University, P.O. Box 1988, Najran 11001, Saudi Arabia; 5 Department of Prosthodontics, Faculty of Stomatology, Yerevan State Medical University after Mkhitar Heratsi, Str. Koryun 2, Yerevan 0025, Armenia; heboyan.artak@gmail.com; 6 Department of Conservative Dentistry & Endodontics, Saveetha Dental College & Hospitals, Saveetha Institute of Medical, Technical Sciences University, Chennai 600077, India; dr.isaq@gmail.com; 7 Department of Restorative Dentistry & Endodontics, Faculty of Dentistry, University of Puthisastra, Phnom Penh 12211, Cambodia

**Keywords:** PEEK, resin cement, tensile bond strength, visio.link adhesive, Zirconia

## Abstract

This in vitro research aimed to evaluate the Tensile Bond Strength of Poly Ether Ether Ketone and Zirconia copings using resin cement with or without Visio.link adhesive. From commercially available Zirconia and PEEK, blocks were machined milled using (CAD)/(CAM) to obtain 20 Zirconia and 20 PEEK copings. These specimens were sandblasted using 110 μm of alumina. The two main groups (20 Zirconia and 20 PEEK copings) were divided further into 4 subgroups, GROUP 1 (*n* = 10) PEEK substructure with self-adhesive resin cement without pretreatment, and GROUP 2 (*n* = 10) PEEK substructure with self-adhesive resin cement pre-treated with Visio.link adhesive. GROUP 3 (*n* = 10) Zirconia copings with self-adhesive resin cement without pretreatment. GROUP 4 (*n* = 10) Zirconia copings with self-adhesive resin cement pre-treated with Visio.link adhesive. Universal testing machine was used to evaluate the tensile bond strength of these copings. The results were analyzed using SPSS software Version 25.0 (SPSS Inc., Chicago, IL, USA). One-way ANOVA and independent *t*-test were used to compare the mean scores. Statistically significant increase was observed in Tensile Bond Strength of samples when Visio.link adhesive was used. Tensile Bond Strength of PEEK copings and Zirconia copings with Visio.link adhesive is considerably greater than PEEK copings and Zirconia copings without adhesive. The mean Tensile Bond Strength of Zirconia (with or without adhesive) is less as compared to Tensile Bond Strength of PEEK (with or without adhesive), but the difference is not statistically significant.

## 1. Introduction

PEEK (Poly Ether Ether Ketone), regarded as methacrylate-free semicrystalline thermoplastic material, is a crucial prototypical member of the polyacryletherketone (PAEK) family [1]. It comprises aromatic benzene molecule along with functional ether or ketone group. The earlier literature on PEEK has shown that it has better chemical, thermal, mechanical, and biological properties in comparison to many restorative materials used today [2]. It eliminates the metallic taste and allergic reaction thus used for fabrication of metal framework in Removable Partial Denture, especially in patients allergic to cobalt chromium framework. It is also used in the fabrication of provisional implant and healing abutments [2,3].

PEEK is advantageous over other materials, however, its whitish color, low translucency, and absence of durable bond limits its use as a restorative material [4]. Hence, to overcome this limitation and enhance viscosity, bond durability and mechanical retention filler content of PEEK was increased [4,5]. The chemical adhesion is another way of achieving durable bond. Studies have shown that, due to low surface energy of PEEK, the bonding between the substructure and the resin cement decreased [4]. According to the study done by Patrick R. Schmidlin, a failure rate of 15% was seen with copings aluminum oxide sand blasted, but only when adhesive was not used [6].

Adhesives improve the bonding by complex exchange between the substructure and resin cement [2]. Higher bond strength can be attained by adhesives with compositions of Methylmethacrylate (MMA) and Dimethacrylate (DMAs) [7].

Zirconia, on the other hand, has proven chemical and dimensional stability. It has opened new horizons for metal-free dentistry in the past few years. It has superior wear resistance in comparison with other ceramic materials [8]. The most common problems associated with zirconia restorations are chipping and debonding. Surface roughness and the type of bonding agent used determine the binding strength of zirconia [9].

Visio.link is a light curing bonding agent (primer) for veneers and artificial teeth based on polymethylmethacrylate and composites. It ensures firm bonding of highly cross-linked PMMA teeth. Researchers have shown that visio.link has the highest bond strength with PEEK restorations when used following ideal protocol and alumina blasting. Visio.link is a bonding agent that incorporates methyl methacrylate monomer. As a result, it is possible that the visio.link dissolved the tested PEEK material at its surface, and the free carbon double bond polymerized with carbon compounds from the bonding agent and the resin composite cement. MMA and PETIA (pentaerythritol triacrylate) are the two primary components of Visio.link. Visio.link gave higher binding strength values to PEEK restorations because of PETIA’s high capacity to change the PEEK surface [10].

Tensile Bond Strength helps us to determine the load at which the adhesive bond of resin cement with coping and tooth fails and dislodgement of coping takes place.

The studies done before were on PEEK material to determine the best adhesive and pre-treatment required to increase the bond strength and thus the durability of the restoration. However, the most commonly used aesthetic material, along with PEEK, is Zirconia. Previous studies have shown that applying a 10-Methacryloyloxydecyl dihydrogen phosphate (MDP)-containing bonding or silane-bonding agent mixture to ceramic zirconia yields superior shear bond strength. In a study done by Hasan Sarfaraj et al. in 2020, 6-methacryloyloxyhexyl phosphonoacetate (6-MHPA) was used as an adhesive agent for zirconia and composite veneer. The good chemical interaction between the 6-MHPA or 6-MHPP (-P(=O)(OH)2) phosphonic acid group and metal oxides on the zirconia surface was assumed to be responsible for the effective zirconia bonding [11]. Previous research by Bonga Stawarczy in 2013 found that increasing the surface area of PEEK using airborne-particle abrasion and using MMA-containing adhesive solutions improves the bonding characteristics [4]. In the present study, we will see the bond strength of zirconia with MMA containing adhesive and the comparison of PEEK and Zirconia tensile bond strength.

The null hypothesis tested was that there are no differences in the Tensile Bond Strength of Poly Ether Ether Ketone and Zirconia using resin cement with or without Visio.link adhesive.

## 2. Materials and Methods

This experimental in vitro study aims to evaluate the Tensile Bond Strength of PEEK and Zirconia copings on freshly extracted maxillary central incisor under universal testing machine before the commencement of study IEC approval was obtained with reference number 2019/IEC/300/3.

The inclusion criteria for the study was freshly extracted maxillary central incisor, and exclusion criteria were posterior tooth, carious tooth, restored tooth, or fractured tooth.

### 2.1. Fabrication of Specimen

**Figure 1:**
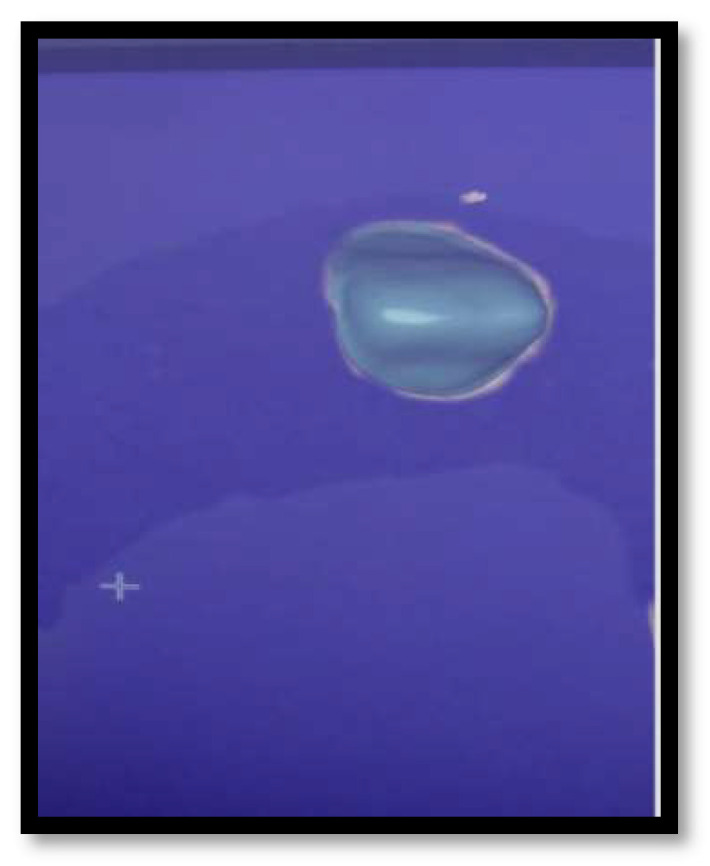
Fabrication of coping in CAD/CAM.

**Figure 2:**
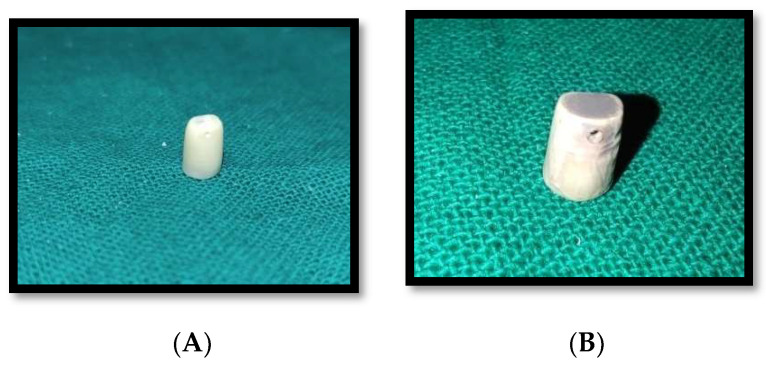
(**A**) PEEK coping, (**B**) Zirconia coping.

**Figure 3:**
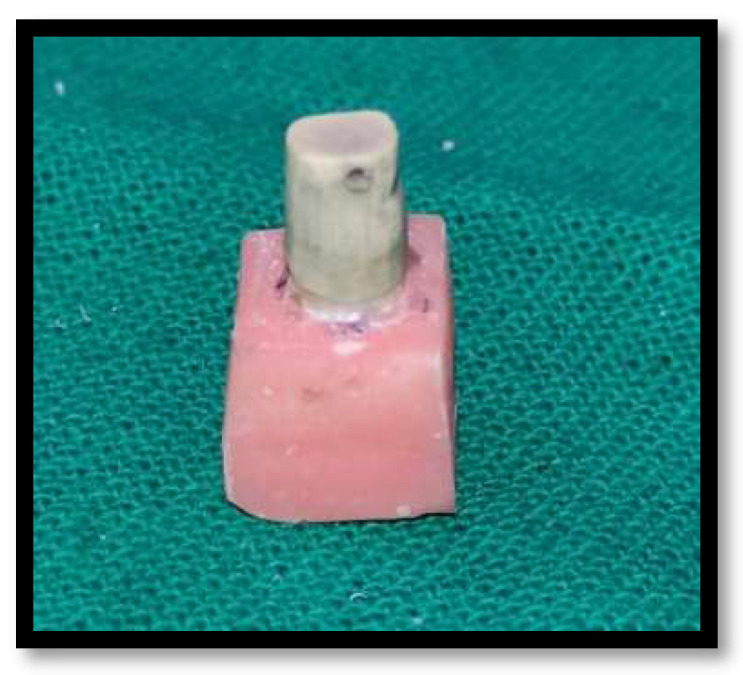
Coping seated on the specimen.

**Figure 4:**
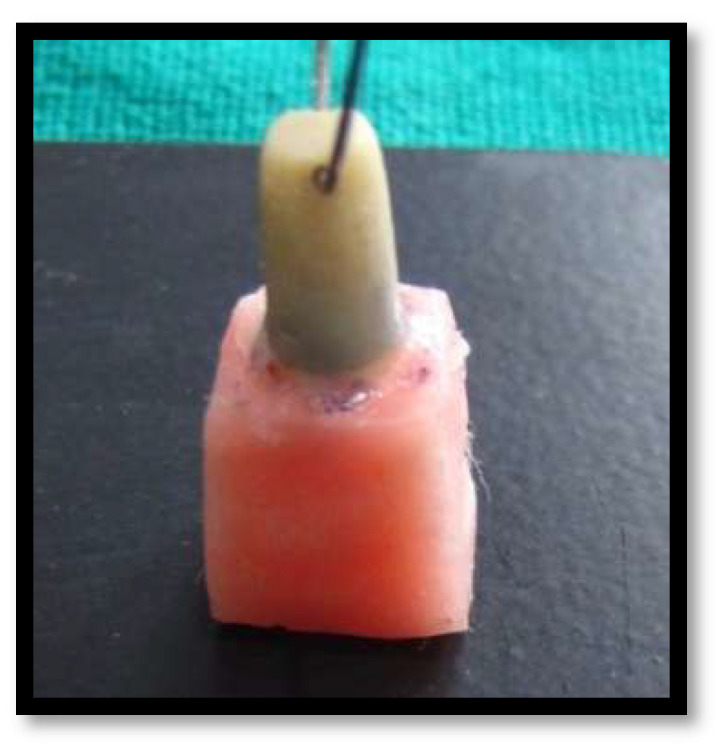
1 mm hole buccolingually below the incisal edge in coping, 23-gauge wire passing through the hole to provide anchorage.

A freshly extracted Maxillary Central Incisor was collected from Oral and Maxillofacial Surgery department. Patient consent was taken, patient was a known case of aggressive periodontitis, and extraction of mobile tooth was done. The calculus deposits and soft tissue debris were washed off using 0.5% sodium hypochlorite and tap water. The natural teeth were used in the study because we needed to gauge the bond strength of peek and zirconia adhesively luted to human dentin. The tooth was then entrenched into a self-cure acrylic resin block. The tooth was prepared under standard dimension for all ceramic crown, and a shoulder gingival finish line of 1 mm width was uniformly made. An impression of prepared tooth was made using addition polyvinylsiloxane material. The impression was then sent to a commercial lab (Kolaj Dental Lab PUNE, Pune, India) for fabrication of PEEK and Zirconia copings using CAD/CAM software (Ceramill®EXO 4.0 CAD, Kolaj Dental Lab, Pune, India) (Figure 1, Figure 2 and Figure 3). The lab was instructed to create a 1 mm hole buccolingually below the incisal edge in all the copings during fabrication. This was done to facilitate the placement of a 23-gauge wire in the hole to provide anchorage of the copings to the upper cross head of universal testing machine (Figure 4).

The specimens were surface treated using sandblasting of 110 μm of aluminum oxide.

The sample consisted of 20 Zirconia and 20 PEEK copings. This sample size was selected because it was the minimum required number to obtain statistically significant result. These groups were further divided depending upon the use of adhesive. The groups are as follows:

Groups surface Treatments

1. GROUP 1 (*n* = 10): PEEK substructure cemented with self-adhesive resin cement using no pre-treatment.
2. GROUP 2 (*n* = 10): PEEK substructure cemented with self-adhesive resin cement pre-treated using visio.link adhesive
3. GROUP 3 (*n* = 10): Zirconia copings cemented with self-adhesive resin cement using no pre-treatment.
4. GROUP 4 (*n* = 10): Zirconia copings cemented with self-adhesive resin cement pre- treated using visio.link adhesive.

Group 1 (*n* = 10):

Resin cement (Rely X Luting2; 3M ESPE) was mixed and manipulated following manufacturer’s instruction and the PEEK copings were light cured for 5 s and cemented.

Group 2 (*n* = 10):

Visio.link adhesive was applied on the PEEK copings for 5 s using brush, then Visio.link was polymerized for 90 s with the help of halogen light curing unit (HAL-LCUs). The polymerization time was chosen according to the manufacturer recommendation. After the application of adhesive, the resin cement (Rely X Luting2; 3M ESPE) was mixed and manipulated according to the manufacturer’s instruction and the PEEK copings were cemented. Cement was light cured for 5 s per side.

Group 3 (*n* = 10):

Resin cement (Rely X Luting2; 3M ESPE) was mixed and manipulated according to the manufacturer’s instruction and the zirconia copings were cemented and light cured for 5 s per side.

Group 4 (*n* = 10):

Visio.link adhesive was applied on the Zirconia copings for 5 s using a brush, then Visio.link was polymerized for 90 s with the help of HAL-LCUs. The polymerization time was chosen according to the manufacturer’s recommendation.

After the application of adhesive, the resin cement (Rely X Luting2; 3M ESPE) was mixed and manipulated according to the manufacturer’s instruction and the Zirconia copings were cemented. Cement was light cured for 5 s per side.

### 2.2. Testing of Specimen for Tensile Bond Strength

Universal Testing Machine (UTM) was employed to evaluate the tensile bond strength of the samples. A 23-gauge wire was attached through the hole for testing the specimens as shown in Figure 4. The machine consists of an upper holding device and lower holding device. The acrylic resin block was fixed in lower holding device and the loop wire attached to the specimen was engaged by upper holding device.

**Figure 5:**
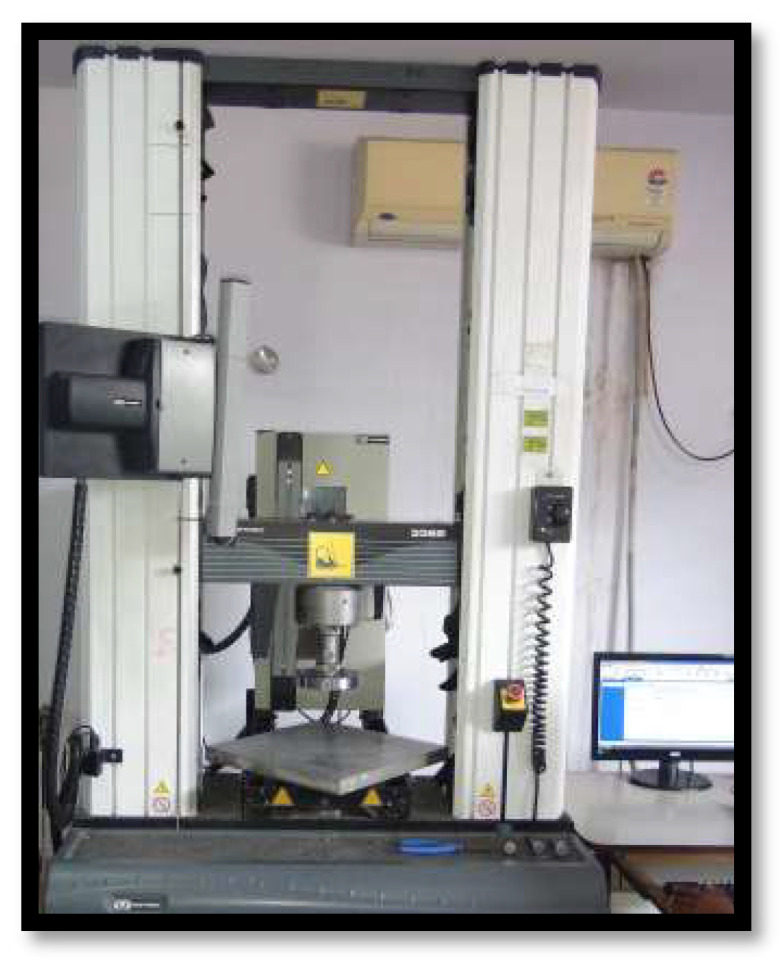
INSTRON 3382 series operating on Bluehill software Universal Testing Machine.

**Figure 6:**
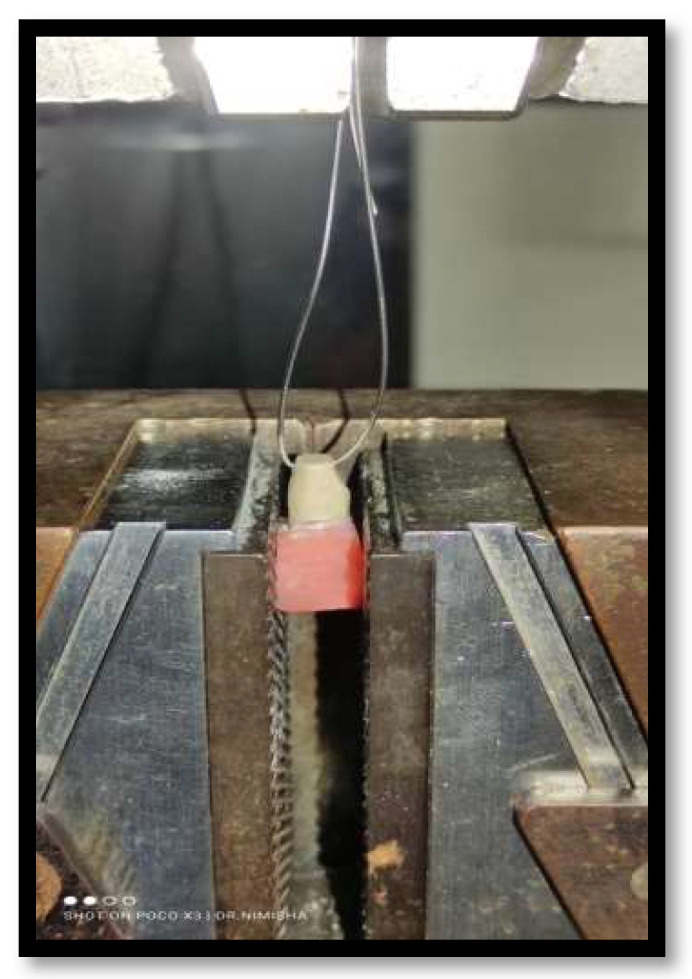
Showing the specimen in Universal Testing Machine.

The universal testing machine (INSTRON 3382 series operating on Bluehill software) with a crosshead speed of 0.5 mm/min was used to dislodge the cemented copings and the force required for the same was measured (Figure 5 and Figure 6).

**Figure 7:**
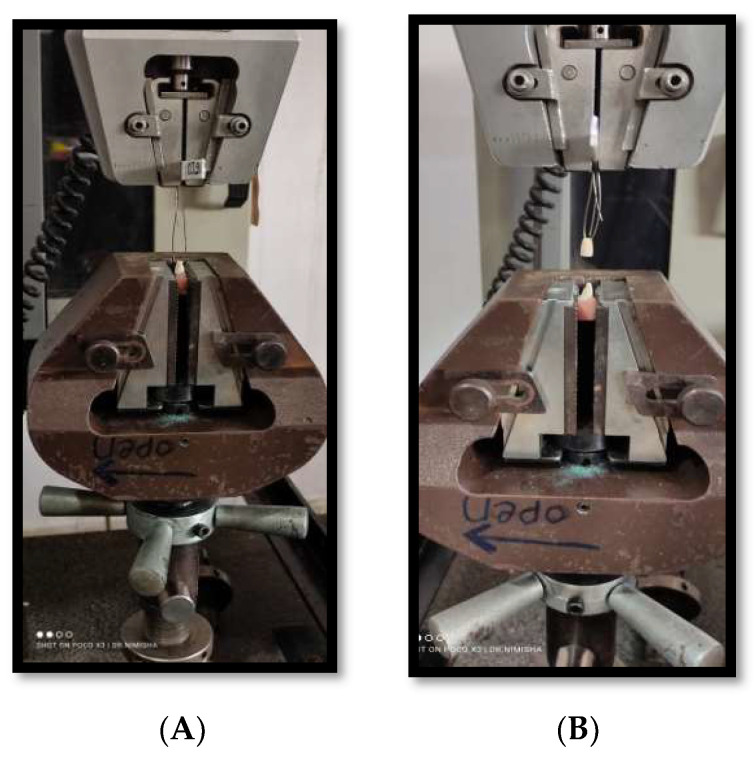
(**A**) Force was applied to dislodge the cemented copings. (**B**) The readings at which copings were debonded were noted.

The readings at which copings were debonded were noted (Figure 7). The dislodging force measured in Newton (N) was changed into MegaPascals (MPa) by dividing it with the surface area (mm^2^) of the prepared tooth. The below-mentioned formula was used to calculate the surface area of tooth.

Area = π/4 d12 + πh/2 (d1 + d2) + π/4 (d32 − d22)

where d1—diameter at the top of the preparation, d2—diameter at the base of the preparation, d3—diameter of the base of the preparation plus 1 mm margins either side, h—axial height.

The dimension of the tooth was measured using Vernier Calliper. The following are the specification:

d1 = 2 mm

d2 = 4.7 mm

h = 7.5 mm

d3 = 5.7 mm

Thus, the surface area calculated was 90.232 mm^2^. The following formula was used to measure the tensile bond strength:

Tensile bond strength (Mpa) = Force (N) × Area (mm^2^)

### 2.3. Statistical Analysis

Data was calculated in Microsoft Excel spreadsheet and descriptive data was evaluated using SPSS software Version 25.0 (SPSS Inc., Chicago, IL, USA). The statistical test one-way ANOVA and independent *t*-test were used to obtain the results.

## 3. Results

The dependent variables used in the study were zirconia, PEEK (a high-performance polymer), resin cement, and Visio.link adhesive.

**Figure 8:**
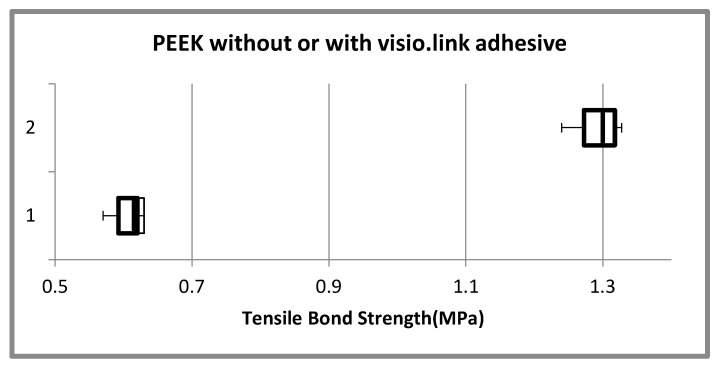
Comparison of mean tensile bond strength of poly ether ether ketone (PEEK) with and without visio.link adhesive. Vertical Axis: 1—PEEK without Visio.link adhesive, 2—PEEK with Visio.link adhesive.

The outcome of the result depicted that the mean tensile bond strength of Poly Ether Ether Ketone (PEEK) without visio.link adhesive group was 0.607 + 0.0211 (MPa) and for PEEK with visio.link adhesive group it was more 1.292 + 0.0282 (MPa), thus the result shows a significant difference between these groups (*p*-0.000) (Figure 8) (Table 1).

**Figure 9:**
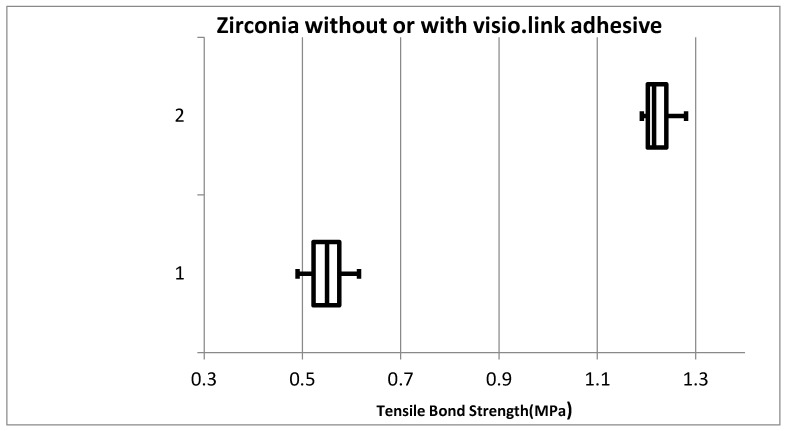
Comparison of mean tensile bond strength of Zirconia with and without visio.link adhesive.Vertical Axis:1—Zirconia without Visio.link adhesive, 2—Zirconia with Visio.link adhesive.

The mean tensile bond strength of Zirconia without visio.link adhesive group was 0.549 + 0.0375 (MPa) and for Zirconia with visio.link adhesive group it was more 1.225 + 0.0317 (MPa) and the difference between the groups was also highly significant (*p*-0.000) (Figure 9) (Table 2).

**Figure 10:**
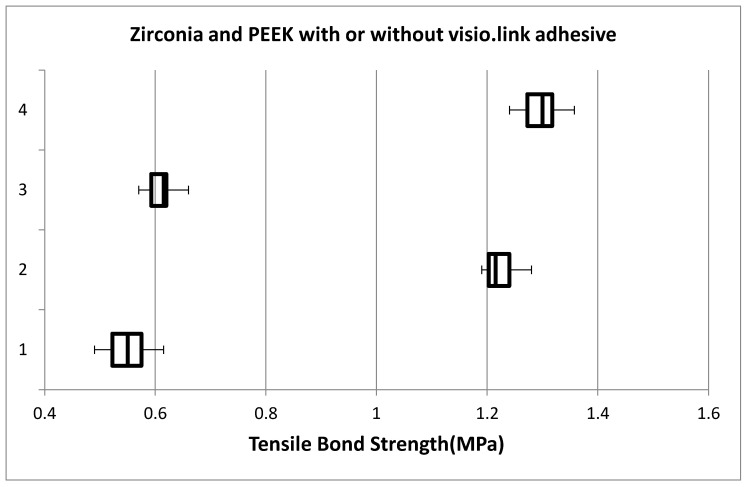
Comparison of mean tensile bond strength of poly ether ether ketone (PEEK) and Zirconia with and without visio.link adhesive.Vertical Axis: 1—Zirconia without Visio.link adhesive, 2—Zirconia with Visio.link adhesive, 3—PEEK without Visio.link adhesive, 4—PEEK with Visio.link adhesive.

The comparative difference in Tensile Bond Strength between Zirconia and PEEKgroup was Zirconia without visio.link adhesive showed 0.549 + 0.0375 (MPa) and mean tensile bond strength of Poly Ether Ether Ketone (PEEK) without visio.link adhesive was 0.607 + 0.0211 (MPa). For PEEK with visio.link adhesive was 1.292 + 0.0282 (MPa), and for Zirconia with visio.link adhesive was 1.225 + 0.0317 (MPa). Both the groups with visio.link adhesive showed more mean tensile bond strength compared to those without visio.link adhesive group, and the result showed a significant difference between these groups (*p*-0.000) (Figure 10) (Table 3).

**Figure 11:**
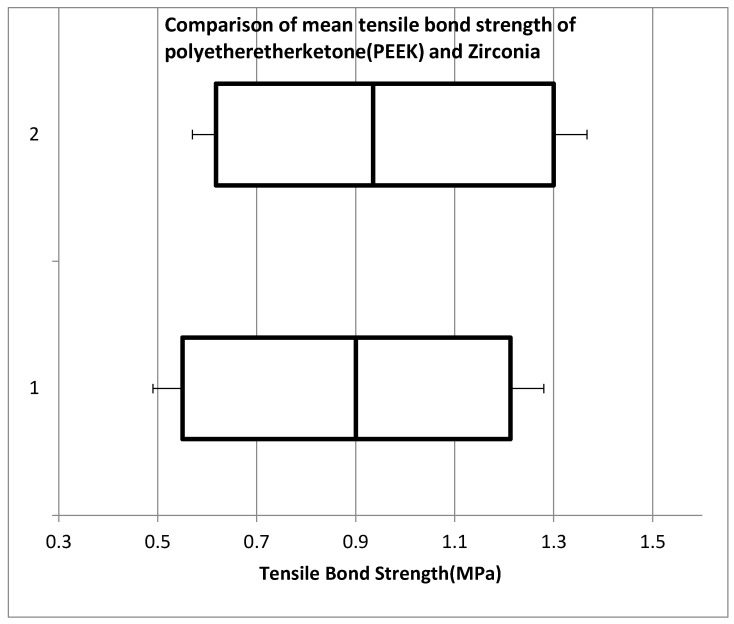
Comparison of mean tensile bond strength of poly ether ether ketone (PEEK) and Zirconia.Vertical Axis: 1—Zirconia with and without Visio.link adhesive, 2—PEEK with and without Visio.link adhesive.

When we compare the PEEK (with and without visio.link adhesive) and zirconia (with and without visio.link adhesive) groups, the mean tensile bond strength of Zirconia was less 0. 887 + 0.3484 compared to PEEK group 0.949 + 0.3522, but the result shows no statistically significant difference (*p*-0.576) (Figure 11) (Table 4).

The obtained mean scores were analyzed using One-way ANOVA and Independent *t*-test. In all the statistical analysis purposes, significant *p*-value was taken ≤0.05.

## 4. Discussion

Owing to increasing demand of aesthetically pleasing dental material, PEEK and Zirconia are the popularly used restorative materials. However, the major shortcoming of PEEK and Zirconia is the absence of durable bond, which must be achieved for long term stability. The reasons for the absence of durable bond could be inadequate preparation of abutment teeth or wrong application technique, but the major reason is absence of bonding agent [12]. Durable bond can be achieved by increasing mechanical retention or by additional chemical adhesion. In this study, pretreatment with 110 µm aluminum oxide particle was done to create roughness on copings surfaces, and thus increase mechanical retention. A study done by Hiroyasu Koizumi et al. states that no effect of surfaces pre-treatment alone can be seen on tensile bond strength of PEEK and Zirconia copings, although use of visio.link adhesive increases the tensile bond strength significantly [13]. Chemical treatment following alumina abrasion significantly improves the bond strength in comparison to chemical treatment without alumina abrasion [14]. The main explanation for increasing the tensile bond strength after air abrasion is roughness that cannot be created by chemical treatment only. The alumina abrasion increases the surface area, which helps to increase the bond strength between two polymers [5]. Secondly, there will be more functional groups by increasing the surface area, so therefore greater crosslinking of polymer [5]. Kern and Lehmann in 2012 studied the bond strength of PEEK and provisional resin using different methods of surface modifications and adhesive system for conditioning. Mean tensile bond strength of 23.2 Mpa with visio.link adhesive and 7.4 MPa without visio.link adhesive when sulfuric acid was taken as pretreatment of PEEK specimen This study results showed the highest values of MMA-containing adhesive systems [15].

The study conducted by Wolfart et al. in 2019 also showed that air abrasion with 110 μm Al_2_O_3_ activated the zirconia surface by increasing the roughness. The tensile bond strength of Zirconia with air abrasion shows a value of 2.71 ± 0.478, whereas when specimen were treated with HF acid they showed a tensile bond strength of 1.41 ± 0.338, and the third group surface treated with Al_2_O_3_ particles followed by 30 µm silica coated Al_2_O_3_ particles showed the value 4.22 ± 0.698. They concluded that air abrasion of the copings and META containing adhesive resin shows an increase in bond strength [16]. Visio.link is made up of MMA (Methyl methacrylate), PETIA (Pentacrythritoltriacrylate), dimethacrylate, and photoinitiator. One of the important factors responsible for increased bond strength between PEEK and veneering resin is MMA monomers [11]. This was also supported by the study of Kern and Lehmann in 2012, in which they concluded that a satisfactory bond with resin varnish was achievable when the surfaces to be cemented were air abraded. Tensile bond strength value 69.0 MPa was calculated when visio.link adhesive was used for PEEK material, which is quite high in comparison with the other adhesives used such as Z-Prime Plus, Ambarino P60, Monobond Plus, and Signum PEEK Bond I+II [17]. Another contributory factor in favor of visio.link, which significantly enhances the bond strength, is the presence of PETIA which highly modifies the PEEK structure [15]. For polymerization of Visio.link adhesive system different light curing units (LCUs) can be used, such as Halogen light curing unit (HAL-LCU) or LED light curing unit for chair side or lab side, respectively. Earlier research shows that PEEK copings cured using halogen light curing unit had higher tensile bond strength than those cured by LED light curing unit. It is basically due to the chemical composition of visio.link photo initiator diphenyl trimethyl benzoyl phosphine oxide requiring certain wavelengths to cure successfully. It shows maximum absorption at 380 nm, and the mentioned wavelength is provided mainly by halogen LCU [18]. In this study, the adhesive visio.link was polymerized using halogen LCU for 90 s to attain durable bonding.

Relyx luting 2 was resin modified with light cure options that shorten cleanup to seconds. This cement was primarily used for Zirconia crowns, metal, and pediatric crowns [19]. For application, mix the paste for 20 s until evenly mixed. The cementation of the prosthesis was done following the guidelines of the manufacturer.

Each specimen was subjected to a debonding force which was recorded using Universal Testing Machine.

The obtained results showed that mean Tensile bond strength of Poly Ether Ether Ketone (PEEK) and Zirconia without visio.link adhesive group was 0.607 + 0.0211 and 0.549 + 0.0375 (MPa), respectively, and for PEEK and Zirconia with visio.link adhesive group was 1.292 + 0.0282 and 1.225 + 0.0317 (MPa), respectively. Both the study groups which used visio.link showed statistically highly significant bond strength (*p*-0.000).

Therefore, we can conclude that after application of adhesive the bond strength of respective copings almost doubles. Therefore, the null hypothesis that there are no differences in the Tensile Bond Strength of Poly Ether Ether Ketone, and Zirconia using resin cement with or without Visio.link adhesive is rejected.

When we compare the PEEK (with and without visio.link adhesive) and zirconia (with and without visio.link adhesive) groups, though the mean tensile bond strength of Zirconia was less 0.887 + 0.3484 compared to PEEK group 0.949 + 03522, the results were not statistically significant (*p*-0.576).

In this article, the tensile bond strength was measured in vitro without thermocycling, and studies should be done after thermocycling for better evaluation of bond strength, although the appropriate and adequate measures were used to control the technicality in the specimen fabrication and to minimize errors while testing.

Future studies should be done to incorporate the composition of Visio.link adhesive to resin cement for better clinical results.

## 5. Clinical Significance

Adhesive helps to build a durable bond of copings with resin cement. The use of resin cement alone is not recommended. In clinical practice we have to include the use of adhesive, and the results will be favorable.

## 6. Conclusions

Bonding of PEEK and Zirconia copings almost doubles after the use of Visio.link adhesive. Adhesives increase the surface area and thus the bonding of copings with natural tooth. Tensile bond strength of PEEK is more than Zirconia, but the difference is not significant.

## Figures and Tables

**Table 1:** Particulars for mean tensile bond strength of PEEK with and without visio.link adhesive.

Particulars	PEEK without Visio.Link Adhesive	PEEK with Visio.Link Adhesive
Minimum	0.57	1.24
Quartile 1	0.59	1.27
Median	0.61	1.30
Quartile 3	0.62	1.31
Maximum	0.63	1.32

**Table 2:** Particulars for mean tensile bond strength of Zirconia with and without visio.link adhesive.

Particulars	Zirconia without Visio.Link Adhesive	Zirconia with Visio.Link Adhesive
Minimum	0.49	1.19
Quartile 1	0.5225	1.2025
Median	0.55	1.215
Quartile 3	0.575	1.24
Maximum	0.61	1.28

**Table 3:** Particulars of mean tensile bond strength of poly ether ether ketone (PEEK) and Zirconia with and without visio.link adhesive.

Particulars	Zirconia without Visio.Link Adhesive	Zirconia with Visio.Link Adhesive	PEEK without Visio.Link Adhesive	PEEK with Visio.Link Adhesive
Minimum	0.49	1.19	0.57	1.24
Quartile 1	0.5225	1.2025	0.5925	1.2725
Median	0.55	1.215	0.615	1.3
Quartile 3	0.575	1.24	0.62	1.3175
Maximum	0.61	1.28	0.63	1.32

**Table 4:** Particulars of mean tensile bond strength of poly ether ether ketone (PEEK) and Zirconia.

Particulars	Zirconia with and without Visio.Link Adhesive	PEEK with and without Visio.Link Adhesive
Minimum	0.49	0.57
Quartile 1	0.55	0.6175
Median	0.9	0.935
Quartile 3	1.2125	1.3
Maximum	1.28	1.32

## Data Availability

The data will be shared upon a reasonable request to the corresponding author.
